# Diagnostic Models of Neonatal Respiratory Distress Syndrome and Congenital Pneumonia: A Retrospective Cohort Study

**DOI:** 10.3390/medsci14030348

**Published:** 2026-06-26

**Authors:** Alfiya Aminova, Anna Zabelich, Bella Matsukatova, Tatyana Eryushova, Kiza Vagidova, Rita Kildiyarova, Albina Polishchuk, Yuliana Osovetskaya, Svetlana Levasheva, Irina Ozerskaia, Olga Sukhovjova, Irina Farber, Svetlana Erdes

**Affiliations:** 1 Department of Propaedeutics of Children’s Diseases, N.F. Filatov Children’s Health Clinical Institute, Sechenov First Moscow State Medical University (Sechenov University), 119991 Moscow, Russia; aminova_a_i@staff.sechenov.ru (A.A.); eryushova_t_yu@staff.sechenov.ru (T.E.); kildiyarova_r_r@staff.sechenov.ru (R.K.); polishchuk_a_r@staff.sechenov.ru (A.P.); erdes_s_i@staff.sechenov.ru (S.E.); 2 N.F. Filatov Children’s Health Clinical Institute, Sechenov First Moscow State Medical University (Sechenov University), 119991 Moscow, Russia; 3 Department of Children’s Diseases, N.F. Filatov Children’s Health Clinical Institute, Sechenov First Moscow State Medical University (Sechenov University), 119991 Moscow, Russia; ozerskaya_i_v@staff.sechenov.ru (I.O.);

**Keywords:** congenital pneumonia, respiratory distress syndrome, neonates, differential diagnosis, models of pathogenetic relationships, predictors, machine learning

## Abstract

**Background**: The differential diagnosis of respiratory distress syndrome (RDS) and congenital pneumonia (CP) in newborns remains a complex clinical challenge due to the similarity in their clinical manifestations and their potential to coexist. **Objective**: We aimed to determine differential diagnostic predictors of RDS and CP in newborns by using mathematical modeling and machine learning methods. **Methods**: A retrospective cohort study was conducted; de-identified medical records of 244 newborns (97 with RDS and 143 with CP) were collected to assess clinical, anamnestic, laboratory, and instrumental data by applying multiple regression analysis, ROC analysis, logistic regression models, and Random Forest. **Results**: Patients with CP presented with a more severe condition at admission (57.1% vs. 23.3%; *p* = 0.023), required mechanical ventilation (MV) more frequently (22.4% vs. 8.2%; *p* = 0.004), and were more often transferred to the intensive care unit (ICU) (77.3% vs. 55.7%; *p* = 0.001). They further had lower hemoglobin levels (151 ± 28 g/L vs. 164 ± 31 g/L; *p* = 0.001) and red blood cell counts (*p* = 0.021). Regression analysis demonstrated that the severity of the condition and the presence of cerebral ischemia were dependent on hemoglobin levels in the case of CP, while gestational age played a determining role in RDS. The machine learning models achieved an accuracy of 0.69 and an area under the curve (AUC) of 0.82 (Random Forest). The key predictors for differential diagnosis of RDS were low gestational age, hyperbilirubinemia, and congenital heart defects, while for CP, they were hemoglobin < 151 g/L, lymphocytes < 4.8 × 10^3^/μL, oxygen saturation < 90–91%, and cerebral ischemia. **Conclusions**: The use of mathematical modeling methods made it possible to identify prognostically significant predictors for the differential diagnosis of RDS and CP. The resulting models are best viewed as proof-of-concept tools for hypothesis generation and future research, as external validation is necessary before they can be considered for clinical use.

## 1. Introduction

Respiratory disorders are the leading cause of intensive care unit hospitalization in neonates and a significant contributor to infant morbidity and mortality worldwide. Neonatal respiratory distress syndrome (RDS) and congenital pneumonia (CP) are the two most common nosological entities responsible for the development of respiratory failure in the first hours and days of life.

RDS is a severe respiratory disorder in preterm neonates caused by morphofunctional immaturity of the lungs and primary surfactant deficiency [1]. As its prevalence is inversely related to birth weight and gestational age, the main risk group consists of preterm infants born before 37–39 weeks [1,2,3,4].

CP is an acute infectious and inflammatory lesion of the lung tissue that typically manifests within the first 48 h of life. Pneumonia is a common infection among preterm newborns undergoing mechanical ventilation [5,6]. In the Russian Federation, the incidence of CP is approximately 1% among full-term newborns and up to 8% among preterm infants born with a birth weight (BW) of 1000 g or more [7]. In children with extremely low birth weight (ELBW), the incidence can reach 25–53.6% [7,8].

### 1.1. Pathogenetic Differences and Clinical Overlap

From a pathogenetic perspective, RDS and pneumonia have fundamentally different natures. RDS results from a primary surfactant deficiency due to the morphofunctional immaturity of type II alveolocytes [9], whereas pneumonia represents an infectious–inflammatory process caused by transplacental, ascending, or intranatal infection [6]. However, despite these etiological differences, both diseases converge through a common final pathway—impairment of pulmonary gas exchange—which accounts for the similarity in their clinical presentation (tachypnea, expiratory grunting, retractions of the compliant areas of the chest wall, and cyanosis) [6,10,11].

### 1.2. Challenges in Differential Diagnosis

The situation is complicated by the following key factors: first, in preterm infants, who constitute the main risk group, clinical and laboratory markers of inflammation are often subtle or nonspecific. Second, the radiological findings in RDS (air bronchograms and reticulogranular pattern) and pneumonia (focal infiltrative opacities) can be nonspecific and overlap, particularly in the early stages. Third, RDS may transform into ventilator-associated pneumonia, or both conditions may coexist, making differential diagnosis not merely challenging but, in some cases, conditional.

### 1.3. Current State of the Problem

A review of the literature from recent years indicates a growing research interest in the search for biomarkers and predictive models for neonatal respiratory pathology. For instance, Alshomrany et al. demonstrated an association between maternal diabetes mellitus and adverse neonatal outcomes [12], Lee et al. showed the role of thyroid dysfunction in the pathogenesis of RDS [13], and Xu et al. identified risk factors for CP in low-birth-weight infants [14]. However, most of these studies are fragmentary, focusing on individual risk factors or outcomes, and fail to propose integrative models suitable for early differential diagnosis.

### 1.4. Scientific Novelty and Significance

In the present study, for the first time, an attempt is made to comprehensively analyze clinical, anamnestic, laboratory, and instrumental data in neonates with RDS and CP by using multiple regression analysis and machine learning methods (i.e., logistic regression and Random Forest). This approach enables not only the identification of statistically significant intergroup differences but also the construction of predictive models with an assessment of their discriminative ability (ROC analysis), as well as the visualization of pathogenetic relationships in the form of mathematical models that have no direct analogs in the available literature.

Thus, the aim of this study is to determine differential diagnostic predictors of RDS and CP in neonates based on mathematical modeling and machine learning methods.

## 2. Materials and Methods

### 2.1. Study Design

A retrospective cohort study was conducted; the medical records of 244 newborns hospitalized in the neonatal pathology unit of a children’s city clinical hospital with RDS or CP were analyzed.

### 2.2. Inclusion Criteria and Group Allocation

This study included patients with a confirmed diagnosis of RDS (*n* = 97) or CP (*n* = 143). The diagnoses were verified based on clinical, radiological, and laboratory data in accordance with current clinical guidelines.

Missing numerical data were imputed using the median, and missing categorical data were imputed using the mode. The proportion of missing data was below 5% for all variables. No other imputation methods were used.

The median age at admission was 4.0 [2.0; 5.0] days: 3.0 [2.0; 5.0] days in the RDS group and 5.0 [3.0; 8.0] days in the CP group.

### 2.3. Clinical and Laboratory Evaluation

A comprehensive examination of patients included assessment of anamnestic data (course of pregnancy and maternal diseases), physical examination with assessment of the severity of the condition, laboratory methods (clinical and biochemical blood tests, blood gas analysis, and bacteriological examination), and instrumental methods (ECG, echocardiography, chest radiography, ultrasound of internal organs, and neurosonography).

### 2.4. Diagnostic Criteria for RDS and CP

All diagnoses of respiratory distress syndrome (RDS) and congenital pneumonia (CP) were established based on a combination of anamnestic, clinical, laboratory, and instrumental criteria, following current clinical guidelines [15,16]. Infants with clinical and radiological evidence of both RDS and CP were excluded from the analysis to ensure clear separation of the two diagnostic groups.

For the RDS group, the following criteria were applied.

The anamnestic criteria were preterm birth, a history of RDS in siblings, maternal gestational or type 1 diabetes mellitus, placental abruption, male fetal sex in preterm delivery, and neonatal asphyxia.

The clinical criteria were dyspnea, expiratory grunting, accessory muscle use, diminished breath sounds, crackles, and cyanosis.

The instrumental (chest X-ray) criteria were reduced lung field area, diffuse decreased lung transparency, progression to “white-out lungs” with indistinct cardiac borders, and an enhanced peripheral bronchial pattern.

The laboratory (arterial blood gas analysis) criteria were respiratory or metabolic acidosis, hypoxemia, hypercapnia, elevated lactate, and hyperglycemia.

For the CP group, the following criteria were applied.

The anamnestic criteria were intrauterine fetal infection, chorioamnionitis, endometritis, the premature rupture of membranes, the aspiration of infected amniotic fluid or vaginal content, maternal infectious–inflammatory diseases (e.g., pyelonephritis and vaginosis), prematurity, birth asphyxia, and fetal hypoxia.

The clinical criteria were thermoregulatory instability (≥38.5 °C or ≤36.0 °C), mottled or grayish skin, perioral or acrocyanosis, decreased tissue turgor, absent or weak sucking reflex, feeding refusal, lethargy or muscle hypotonia, tachycardia or bradycardia (<110 bpm), muffled heart sounds, abdominal distension, and signs of respiratory failure including decreased oxygen saturation, tachypnea, apnea, expiratory grunting, diminished breath sounds, localized fine crackles or crepitation, and reduced response to respiratory support.

The instrumental (chest X-ray) criterion was the presence of infiltrative or focal opacities.

The laboratory criteria were leukocytosis or leukopenia, neutrophilia or neutropenia, elevated erythrocyte sedimentation rate, elevated C-reactive protein (CRP) and procalcitonin, positive microbiological cultures (blood or tracheal aspirate), and arterial blood gas analysis showing hypoxemia, hypercapnia, and metabolic acidosis.

The severity of both respiratory distress and congenital pneumonia was assessed using the Silverman–Andersen score [17].

### 2.5. Ethical Aspects

This study was conducted in accordance with the ethical principles for medical research outlined in the Declaration of Helsinki and was approved by the Ethics Committee of G.N. Speransky Children’s City Clinical Hospital No. 9 (protocol No. 50 dated 14 February 2023).

### 2.6. Statistical Analysis

Data processing was performed using StatTech 4.14.2 (LLC “Stattech”, Russia, 2026). Quantitative variables were tested for the normality of distribution using the Shapiro–Wilk and Kolmogorov–Smirnov tests. Depending on the distribution type, data are presented as the mean (M) ± standard deviation (SD) with 95% confidence interval (CI) or as the median (Me) and interquartile range [Q1; Q3]. Categorical variables are described as absolute values and percentages with 95% CIs (Clopper–Pearson method).

For intergroup comparisons, the following tests were used: Student’s t-test (for normally distributed data), the Mann–Whitney U test (for non-normally distributed data), Pearson’s chi-squared test, or Fisher’s exact test (for categorical variables).

The strength and direction of associations were assessed using the Spearman rank correlation coefficient and Cramer’s V coefficient. To quantify the effect size, the odds ratio (OR) with the 95% CI was calculated.

Predictive models were constructed using binary logistic regression and Random Forest. The discriminative ability of the models was evaluated using ROC analysis with the calculation of the area under the curve (AUC) and the determination of the optimal cutoff point based on Youden’s index. Differences were considered statistically significant at *p* < 0.05.

To account for multiple univariable comparisons (e.g., intergroup comparisons of clinical, laboratory, and instrumental variables), we applied the Benjamini–Hochberg false discovery rate (FDR) correction. Adjusted *p*-values below 0.05 were considered statistically significant. For multivariable regression models, we prioritized model stability using Firth’s penalized logistic regression and bootstrap validation rather than simple *p*-value adjustments.

### 2.7. Machine Learning Procedures

All machine learning analyses were performed using Python 3.8 with the scikit-learn library (version 0.24.2).

Data splitting. The dataset was randomly split into training (80%) and test (20%) subsets by using stratified sampling based on the target variable (diagnosis: RDS vs. CP). Stratification was performed using StratifiedShuffleSplit to preserve the class distribution in both subsets. The test set was held out from the very beginning and was used only for the final evaluation of model performance; it was not involved in any stage of model training or hyperparameter tuning.

Cross-validation and hyperparameter tuning. Hyperparameter optimization was carried out using 5-fold stratified cross-validation (GridSearchCV) on the training set only. The following hyperparameters were tuned:

1. Logistic Regression: Regularization strength (C = 0.01, 0.1, 1, 10, 100) and penalty (L1, L2).
2. Random Forest: Number of trees (n_estimators = 50, 100, 200), maximum depth (max_depth = 5, 10, 20, None), and minimum samples split (min_samples_split = 2, 5, 10).

The best hyperparameter combination was selected based on the highest cross-validated ROC-AUC. The final model was then retrained on the full training set using the selected hyperparameters and evaluated on the independent test set.

Feature selection. Initial candidate variables (*n* = 37) were reduced using a filter method: Univariate statistical tests (the Mann–Whitney U test for continuous variables and the chi-squared test for categorical variables) were applied to compare RDS and CP groups. Only variables with *p* < 0.05 were retained for model development. This resulted in 10 key variables (LAR, lactate, pH, age, PaO_2_/FiO_2_, albumin, BMI, total protein, prothrombin time, and direct bilirubin). These 10 variables served as the candidate pool for subsequent multivariable modeling. The final predictors retained in the penalized logistic regression and Random Forest models were selected from this candidate set using model-specific procedures. For the Random Forest model, embedded feature importance (Gini importance) was additionally used to rank predictors; the top four contributors (hemoglobin, oxygen saturation, birth weight, and gestational age) were confirmed as the most important.

Evaluation metrics. Model performance on the test set was assessed using accuracy, sensitivity (recall), specificity, precision, F1-score, ROC-AUC, and Brier score. The calibration of the logistic regression model was evaluated using a calibration curve (Hosmer–Lemeshow goodness-of-fit test). All metrics are reported with 95% confidence intervals where applicable.

## 3. Results

### 3.1. General Characteristics of the Study Groups

This study included 244 newborns: 97 with RDS and 143 with CP. The gestational age in the RDS group was significantly lower than that in the CP group (median of 36 [33.00; 37.00] weeks vs. 37 [34.00; 38.00] weeks; *p* = 0.015). Among the patients, there were 126 males and 114 females. In the CP group, boys predominated (62.2%), whereas in the RDS group, the sex ratio was equal (50%).

### 3.2. Clinical Characteristics and Disease Severity

Patients with CP required transfer to the intensive care unit more often (77.3% vs. 55.7%; *p* = 0.001) and required mechanical ventilation (MV) more frequently (22.4% vs. 8.2%; *p* = 0.004), reflecting greater disease severity.

**Figure 1:**
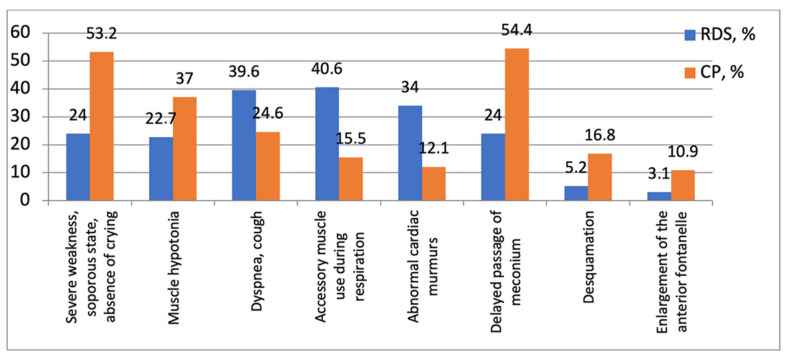
Statistically significant differences in physical examination findings between the study groups.

During clinical assessment, newborns with CP typically presented with marked lethargy, muscular hypotonia, signs of cerebral depression, a higher rate of cerebral ischemia (66.4% vs. 49.5%; *p* = 0.007), and metabolic disturbances (16.8% vs. 8.1%; *p* = 0.049). In children with RDS, dyspnea and the use of accessory respiratory muscles were observed more frequently. A severe condition at admission was significantly more often diagnosed in CP (57.1% vs. 23.3%; *p* = 0.023) (Figure 1).

### 3.3. Laboratory Predictors

Newborns with CP showed lower hemoglobin levels (151 ± 28 g/L vs. 164 ± 31 g/L; *p* = 0.001) and red blood cell counts (*p* = 0.021), as well as changes in the leukocyte formula indicative of inflammation: increased lymphocyte counts (4.80 [3.43; 6.73] × 10^3^/μL vs. 4.08 [3.20; 5.15] × 10^3^/μL; *p* = 0.012) and basophil counts (*p* = 0.021).

In the RDS group, higher levels of total bilirubin (89.88 ± 89.33 μmol/L vs. 155.13 ± 90.54 μmol/L; *p* = 0.004) and AST (36.00 [31.00; 49.75] U/L vs. 32.00 [22.00; 46.00] U/L; *p* = 0.008) were recorded, which is likely related to a lower gestational age and a higher incidence of neonatal hyperbilirubinemia.

However, after adjustment for gestational age and birth weight in a multivariable logistic regression model, neither AST (*p* = 0.134) nor total bilirubin (*p* = 0.097) remained independently associated with the diagnosis of RDS, indicating that these differences are largely explained by the lower gestational age of infants in the RDS group.

Blood gas analysis parameters demonstrated intergroup differences: In CP, a tendency toward hypercapnia was observed (pCO_2_ of 39.41 ± 9.93 mmHg vs. 35.89 ± 8.49 mmHg; *p* = 0.049), reflecting a respiratory component of respiratory failure, whereas oxygen saturation was lower in the RDS group (*p* = 0.004) (Table 1). Although the median SpO_2_ values were identical (98% in both groups), the interquartile range was higher in the CP group, and the difference between the distributions was statistically significant (*p* = 0.004). The absolute difference in SpO_2_ between the groups was small; nevertheless, this variable contributed to the performance of the machine learning models when combined with other predictors.

After applying Benjamini–Hochberg false discovery rate (FDR) correction for multiple univariable comparisons (a total of 14 tests), all the variables listed in Table 1 (hemoglobin, red blood cells, basophils, lymphocytes, SpO_2_, pCO_2_, total bilirubin, and AST), as well as gestational age, transfer to NICU, cerebral ischemia, severe condition at admission, metabolic disturbances, and maternal infections, remained statistically significant (FDR-adjusted *p* < 0.05, q = 0.0014 for all).

### 3.4. Instrumental Data

**Figure 2:**
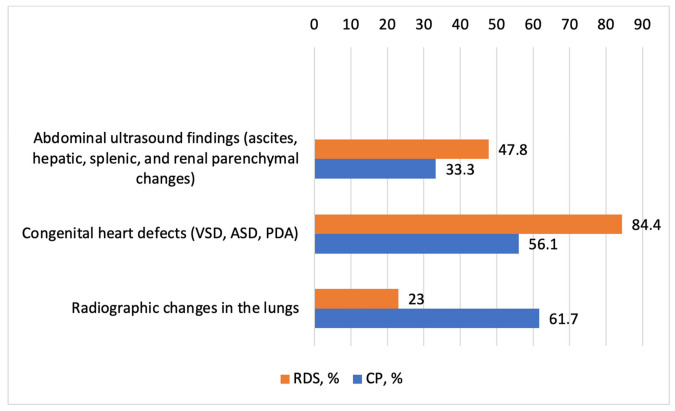
Statistically significant differences in instrumental research data between the study groups. VSD—ventricular septal defect; ASD—atrial septal defect; PDA—patent ductus arteriosus.

According to ultrasound data, patients with RDS had a 1.5-fold higher incidence of congenital heart defects and hydroperitoneum. Cerebral ischemia in RDS occurred 1.9 times less frequently than in CP (49.5% and 66.4%, respectively; *p* = 0.007; OR for absence of pathology = 0.21; 95% CI: 0.07–0.61). Pulmonary changes on chest radiographs were documented in 61.7% of CP cases (Figure 2).

### 3.5. Perinatal Factors and Maternal History

Comorbid gastrointestinal diseases, endocrine system disorders, past infections, and exposure to measles, hepatitis, and other infections in the mother during pregnancy were recorded more frequently in the RDS group (*p* = 0.025, *p* = 0.001, and *p* = 0.023, respectively).

### 3.6. Role of Surfactant Therapy

The frequency of surfactant administration did not differ significantly between the groups (60% in CP vs. 40% in RDS; *p* = 0.881). However, the median gestational age of children receiving surfactant was lower in the CP group—28 weeks [22.00; 36.00] vs. 33 weeks [28.00; 36.00] in the RDS group. An association was found between surfactant administration in children with CP and a more severe course: these patients exhibited pronounced symptoms of multiple organ failure (*p* = 0.028) and cerebral depression (*p* = 0.003).

### 3.7. Regression Analysis and Association Patterns

Congenital Pneumonia. The results of the multiple regression analysis revealed statistically significant correlations among clinical, laboratory, and instrumental parameters (Table 2). Of key importance was the relationship between the hemoglobin level and the degree of hypoxia, disease severity, the depression of reflex and motor activity, and hypoxic–ischemic CNS injury. Decreased hemoglobin was associated with cerebral depression (OR = 6.59; 95% CI: 1.893–22.950; *p* = 0.001) and pathological changes on neurosonography (OR = 4.03; 95% CI: 1.592–10.235; *p* = 0.002).

Respiratory Distress Syndrome. In the RDS group, significantly fewer statistically significant correlations were identified compared with the CP group (Table 3). The condition of patients with RDS was primarily influenced by gestational age (median of 35.00 [34.00; 36.00] weeks for patients with retinal changes; *p* = 0.001), morphofunctional immaturity, and cerebral ischemia (OR = 2.302; 95% CI: 1.021–5.190; *p* = 0.043).

### 3.8. Predictors for Differential Diagnosis (Machine Learning)

Statistically significant differences between the groups were observed for several clinical and laboratory parameters, as detailed in Table 1, Table 2 and Table 3.

Due to small subgroup sizes, some odds ratios (e.g., for mechanical ventilation) were excessively large and unstable. To obtain stable estimates for key predictors, we additionally performed Firth’s penalized logistic regression. As shown in Table 4, lower gestational age was associated with reduced odds of CP (OR = 0.70; 95% CI: 0.60–0.82; *p* < 0.001), while cerebral ischemia remained a strong independent predictor (OR = 3.16; 95% CI: 1.50–6.67; *p* = 0.002). Higher hemoglobin was also associated with reduced odds of CP (OR = 0.98; 95% CI: 0.96–0.99; *p* = 0.015).

Based on the final selected predictors (Table 4), two classification models were constructed:

- Firth’s penalized logistic regression, which achieved an accuracy of 0.66 and an AUC of 0.76 (95% CI: 0.68–0.84);
- Random Forest, which achieved an accuracy of 0.69 and an AUC of 0.82.

Table 5 summarizes the performance metrics of both models on the independent test set (20% holdout). The Random Forest model achieved an ROC-AUC of 0.82, indicating acceptable discriminative ability, but the overall accuracy remained moderate (0.69). The greatest feature importance was observed for hemoglobin, oxygen saturation, birth weight, and gestational age.

### 3.9. Mathematical Models of Pathogenetic Relationships

**Figure 3:**
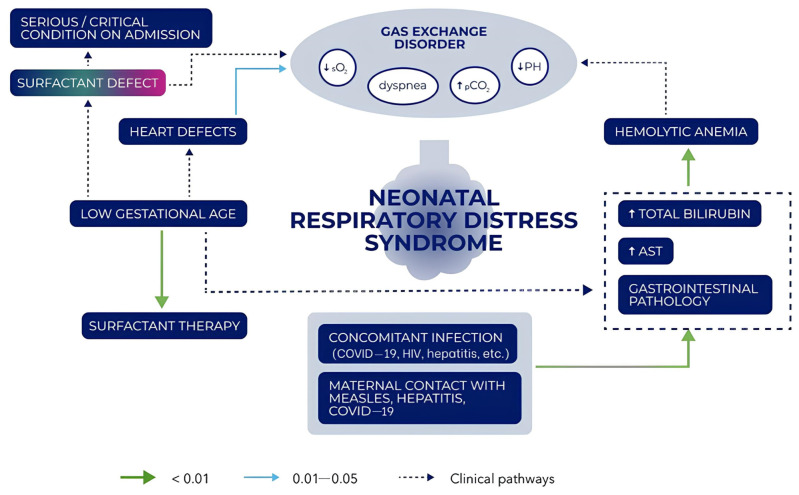
Graphical association model of pathogenetic relationships in respiratory distress syndrome. Solid green arrows indicate strong statistically significant associations (*p* < 0.01); solid blue arrows indicate moderate statistically significant associations (0.01 ≤ *p* < 0.05); dashed blue arrows represent clinically recognized pathways that did not reach statistical significance in our regression models but are conceptually important. Abbreviations: AST—aspartate aminotransferase; COVID—coronavirus disease; HIV—human immunodeficiency virus.

**Figure 4:**
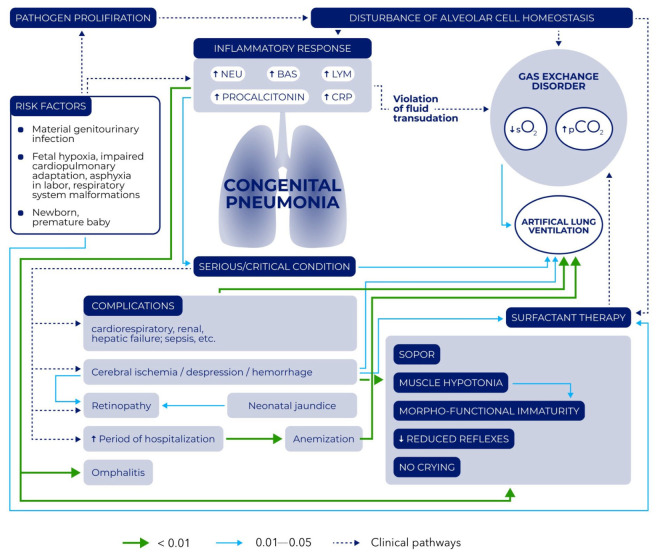
Graphical association model of pathogenetic relationships in congenital pneumonia. Solid green arrows indicate strong statistically significant associations (*p* < 0.01); solid blue arrows indicate moderate statistically significant associations (0.01 ≤ *p* < 0.05); dashed blue arrows represent clinically recognized pathways that did not reach statistical significance in our regression models but are conceptually important. Abbreviations: BAS—basophils; CRP—C-reactive protein; LYM—lymphocytes; NEU—neutrophils; PROCAL—procalcitonin.

Figure 3 and Figure 4 present the mathematical models developed within the framework of this study reflecting the pathogenetic processes associated with the clinical and diagnostic manifestations of each nosological form. In Figure 3 and Figure 4, solid arrows indicate statistically significant associations (*p* < 0.05), whereas dashed arrows represent clinically recognized relationships that are conceptually important but did not reach statistical significance in our regression models.

In RDS, the key pathogenetic link is impaired gas exchange, leading to metabolic acidosis against the background of surfactant system immaturity, the breakdown of fetal hemoglobin with hyperbilirubinemia, and congenital heart defects. An influence of maternal diseases on the degree of hemoglobin reduction was established (Figure 3).

In CP, the main pathogenetic components are the inflammatory process and respiratory acidosis occurring against the background of perinatal risk factors. Predictors of disease development include mechanical ventilation and surfactant administration in very preterm infants. Hemoglobin level (less than 151 g/L), gestational age, and birth weight were established as differential diagnostic predictors (Figure 4).

## 4. Discussion

### 4.1. Epidemiological Characteristics of the Cohort

The present study demonstrated that CP remains a significant cause of neonatal pathology and mortality. In our cohort, mortality due to CP was 2% (3 cases out of 143 patients), which corresponds to the lower limit of the range reported in official data from the Russian Federation (0.35–8.34%) [18] and is substantially lower than global rates (10% of total childhood mortality) [19]. Such a favorable outcome is likely related to modern respiratory support protocols and timely antibiotic therapy in a specialized hospital setting.

A predominance of males was observed in the AP group (62.2%), whereas the sex ratio in the RDS group was equal (50%). This finding is consistent with the observations by Ghafoor T. et al. [4,20] and may be explained by the protective effect of estrogens, which stimulate alveologenesis and surfactant synthesis in females [21]. The results obtained support the hypothesis of greater vulnerability of males to infectious and inflammatory lesions of the respiratory tract in the neonatal period.

### 4.2. Disease Severity and Determining Factors

The clinical assessment of newborns revealed a significantly more severe condition at admission in children with CP (57.1% vs. 23.3% in RDS; *p* = 0.023), which correlated with a higher need for ICU transfer (77.3% vs. 55.7%; *p* = 0.001) and mechanical ventilation (22.4% vs. 8.2%; *p* = 0.004).

Predictably, the factors that aggravated the course of CP were cerebral depression, ischemia, intracranial hemorrhages, and low gestational age. These data are fully consistent with the current understanding of the comorbidity of neonatal pathology: the more immature the newborn, the higher the likelihood of a systemic response to a local inflammatory process involving the central nervous system [22,23].

### 4.3. Perinatal Risk Factors

The analysis of the maternal history revealed that in the RDS group, gastrointestinal diseases, endocrine system disorders, and past infections, as well as exposure to measles, hepatitis, and other infections, were recorded significantly more frequently (*p* < 0.05). These data correlate with those of other studies [12,13,14,24]. Maternal diabetes mellitus, according to the study by Alshomrany A. et al., increases the risk of adverse neonatal outcomes (aOR = 1.46; 95% CI: 1.25–1.70), which is likely related to fetal hyperinsulinemia, which slows the maturation of the surfactant system [12]. Thyroid dysfunction (elevated TSH > 4 mIU/L) is associated with a 2.83-fold increased risk of RDS [13], which may be attributed to the effect of thyroid hormones on type II pneumocyte differentiation. Maternal infectious diseases can induce preterm birth, which is a leading risk factor for RDS, or cause the transplacental transmission of the pathogen leading to CP [14,24].

### 4.4. Differential Diagnostic Predictors

The search for differential diagnostic criteria for CP and RDS represents a complex clinical challenge, as both the transformation of RDS into CP (due to complications associated with mechanical ventilation) and their combination are possible [24]. Aleksandrovich Yu.S. et al., in a study of 180 newborns with RDS, diagnosed congenital pneumonia in 3% of cases, which underscores the need for careful differential diagnosis [25].

Researchers have made numerous attempts to assess the probability of developing CP in the setting of RDS. Based on the results of our study, we identified the risk factors of maternal infection (urinary tract), fetal hypoxia, birth asphyxia, and low gestational age. These findings are consistent with the literature [14].

In our study, the median gestational age of children who received surfactant was 28 weeks [22.00; 36.00] in the CP group and 33 weeks [28.00; 36.00] in the RDS group, which is consistent with published data [25]. Children with CP who received surfactant had a lower gestational age and a more severe clinical course (including multiple organ failure and cerebral depression). It is likely explained by confounding factors: lower gestational age is independently associated with both a higher likelihood of surfactant administration and a higher risk of infection. Several authors have hypothesized that surfactant administration might be associated with early airway infection in very preterm infants, but this remains unproven [26,27,28,29].

Importantly, high-quality evidence from Cochrane systematic reviews confirms that surfactant administration in preterm infants with RDS significantly reduces mortality and the incidence of pneumothorax without increasing the risk of infectious complications [30,31].

Therefore, the observed association between surfactant therapy and CP in our cohort most likely reflects the underlying vulnerability of extremely preterm infants rather than a direct adverse effect of the drug itself.

### 4.5. Laboratory and Instrumental Predictors

The results of the multiple regression analysis allowed us to identify statistically significant correlations with potential pathophysiological relevance. In the CP group, the relationship between the hemoglobin level and the degree of hypoxia, disease severity, depression of reflex activity, and hypoxic–ischemic CNS injury was of key importance (95% CI: 8.38–34.15; *p* = 0.001). Anemia in newborns with CP likely reflects the myelotoxic effect of the infectious agent, a hemodilution component in the setting of massive fluid therapy, and increased erythrocyte destruction under conditions of systemic inflammation.

In the RDS group, the condition of patients was primarily influenced by gestational age ([34.00; 36.00], *p* = 0.001), morphofunctional immaturity ([34.00; 36.00], *p* = 0.001), and cerebral ischemia (95% CI: 1.021–5.190; *p* = 0.043). Notably, the number of statistically significant correlations in RDS was substantially lower than in CP, reflecting the more monofactorial nature of RDS pathogenesis (where surfactant deficiency is the leading component) compared with the multifactorial pathogenesis of CP.

### 4.6. Comparison with Current Prognostic Models

The mathematical models we developed (Figure 3 and Figure 4) represent a graphical visualization of the pathogenetic processes associated with the clinical and diagnostic manifestations of RDS and CP. In the literature available to us, no analogs of such a structured representation indicating the statistical significance of the relationships were found.

Contemporary research in this field is evolving in a similar direction. Goryachko A.N. et al. presented a predictive model for CP based on pregnancy complications, a history of adverse obstetric outcomes, and severe respiratory failure [32]. Ari M. et al. demonstrated the diagnostic significance of hematological and inflammatory biomarkers in predicting mortality in children with severe pneumonia [33]. Cao S. et al. identified risk factors for adverse outcomes (oxygen saturation, hemoglobin, lipase, and urea) and constructed a predictive model for personalized risk assessment [34]. Lei Y. et al., in a cohort of 2705 preterm infants, developed a nomogram for predicting respiratory failure in RDS that included gestational age < 28 weeks, blood gas parameters, hemoglobin level, and the use of respiratory support [35]. Baseer K.A.A. et al. confirmed the leading role of preterm birth, prematurity, and maternal diabetes mellitus in the structure of risk factors for RDS and CP [36]. Lv J. et al. developed a machine learning-based predictive model to assess 28-day mortality in adult patients with pneumonia complicated by RDS [37]. Their study identified the 10 most prognostically significant clinical variables: lactate-to-albumin ratio, lactate, blood pH, patient age, oxygenation index, albumin, body mass index, total protein, prothrombin time, and direct bilirubin.

### 4.7. Clinical Significance and Prospects for Application

The data obtained in our study allow us to formulate practical recommendations for differential diagnosis. The priority predictors of RDS include low gestational age, hyperbilirubinemia (which largely reflects prematurity), congenital heart defects, concomitant gastrointestinal pathology, and infectious diseases in the mother. The priority predictors of CP include a hemoglobin level < 151 g/L, lymphocytes < 4.8 × 10^3^/μL, oxygen saturation < 90–91%, concomitant cerebral ischemia, and inflammatory changes in the leukocyte formula.

The models developed using machine learning methods (Firth’s penalized logistic regression, with an accuracy of 0.66 and an AUC of 0.76, and Random Forest with an accuracy of 0.69 and an AUC of 0.82) can serve as a foundation for creating a risk calculator suitable for use in clinical practice. Hemoglobin, oxygen saturation, birth weight, and gestational age demonstrated the greatest prognostic significance.

It is important to note that while oxygen saturation (SpO_2_) was statistically different between the groups and contributed to the machine learning model’s performance, the absolute difference was small. In clinical practice, SpO_2_ should not be used as a standalone diagnostic test for differentiating RDS from CP but rather as one component of a multivariate assessment.

Similarly, while total bilirubin levels were significantly higher in the RDS group in the univariable analysis (*p* = 0.004), this difference lost statistical significance after adjustment for gestational age and birth weight (*p* = 0.097). Therefore, hyperbilirubinemia in RDS largely reflects gestational immaturity rather than representing an independent disease-specific diagnostic marker. Clinicians should interpret elevated bilirubin in the context of the infant’s gestational age.

Given the moderate predictive performance of our models (accuracy of 0.66–0.69 and sensitivity of 0.60–0.62), these findings should be considered exploratory and hypothesis-generating. External validation in larger, multicenter cohorts is necessary before any clinical implementation can be recommended.

### 4.8. Study Limitations

When interpreting the results, several limitations should be taken into account, such as the retrospective design of this single-center study, the relatively small sample size for machine learning, and the possible influence of unaccounted factors (e.g., genetic polymorphisms and nosocomial infection).

Furthermore, despite our adherence to national clinical guidelines for the diagnosis and management of RDS and CP [15,16], local diagnostic and treatment practices may have influenced the findings. This may limit the generalizability of our results to other institutions with different resources or diagnostic algorithms. Accordingly, multicenter prospective studies are needed to validate our findings across diverse clinical settings.

Despite the use of explicit clinical and radiological criteria to assign patients to diagnostic groups, the substantial clinical and radiological overlap between RDS and CP means that some degree of misclassification cannot be completely excluded in a retrospective study. This is an inherent limitation of any study comparing these two conditions.

Another limitation concerns the potential confounding effect of gestational age. Ideally, we would have performed stratified analyses by gestational age subgroups (e.g., extremely preterm, moderate-to-late preterm, and term) to assess whether the identified predictors remained consistent across different maturity levels. However, due to the very small number of extremely preterm infants in our cohort (*n* = 4 in total: 2 with RDS and 2 with CP) and the imbalance in sample sizes across strata, such subgroup analyses were not statistically feasible. Future large-scale multicenter studies are needed to validate our findings in specific gestational age subgroups, particularly among extremely preterm infants.

Additionally, some odds ratios reported in Table 2 (e.g., for mechanical ventilation in CP) were excessively large, with very wide 95% confidence intervals. This indicates model instability, likely due to small numbers of patients in certain subgroups (rare events) and collinearity among predictors (e.g., gestational age and birth weight). To address this, we applied Firth’s penalized logistic regression and bootstrap validation, which produced more stable estimates. Readers should interpret the magnitude of the original ORs with caution, and future studies with larger sample sizes are needed to confirm these associations.

The machine learning models developed in this study demonstrated only moderate predictive performance (accuracy of 0.65–0.69 and sensitivity of 0.60–0.62). While the Random Forest model achieved an acceptable AUC of 0.82, accuracy and sensitivity are insufficient for standalone clinical implementation. Therefore, these models should be considered proof-of-concept tools for risk stratification. Future studies with larger sample sizes, multicenter data, and additional predictors are needed to improve performance.

## 5. Conclusions

This study confirmed the complexity of the differential diagnosis of respiratory distress syndrome (RDS) and congenital pneumonia (CP) in newborns, ascribed to both the similarity in their clinical presentation and their potential to coexist.

Using mathematical modeling and machine learning methods, we identified statistically significant predictors that may assist in early risk stratification, pending further validation. For RDS, the key predictors were low gestational age, hyperbilirubinemia (which largely reflects prematurity), and congenital heart defects. For CP, the key predictors were hemoglobin < 151 g/L, lymphocytes < 4.8 × 10^3^/μL, oxygen saturation < 90–91%, and cerebral ischemia.

After adjustment for gestational age and birth weight, elevated AST and total bilirubin levels were no longer independently associated with RDS, indicating that these laboratory differences are largely explained by prematurity rather than representing disease-specific markers.

The machine learning models developed in this study (Firth’s penalized logistic regression, with an accuracy of 0.66 and an AUC of 0.76; Random Forest, with an accuracy of 0.69 and an AUC of 0.82) demonstrated moderate performance. While the Random Forest model showed acceptable discriminative ability (AUC = 0.82), the accuracy and sensitivity remain insufficient for standalone clinical implementation. Therefore, these models should be considered proof-of-concept tools for risk stratification.

A promising direction is the development of mobile applications and risk calculators based on the obtained models. Future multicenter studies with larger sample sizes are needed to validate these findings and improve model performance.

## Figures and Tables

**Table 1:** Statistically significant differences in laboratory findings between the study groups.

Parameters	RDS		CP		*p*-Value
	Me	Q1; Q3	Me	Q1; Q3	
Hemoglobin, g/dL	164.87 ± 31.26	158.60; 171.14	151.04 ± 28.51	146.33; 155.75	*p* = 0.001 *
RBC, 10^12^/L	4.64	4.47; 4.80	4.38	4.24; 4.52	*p* = 0.021 *
Basophils, 10^3^/µL	0.07	0.04; 0.129	0.10	0.06; 0.18	*p* = 0.021 *
Lymphocytes, 10^3^/µL	4.08	3.20; 5.15	4.80	3.43; 6.73	*p* = 0.012 *
Saturation peripheral oxygen (SpO_2_), %	98.00	97.00; 98.25	98.00	98.00; 99.00	*p* = 0.004 *
Partial pressure of carbon dioxide (pCO_2_), mmHg	M 35.89	SD 8.49	M 39.41	SD 9.93	*p* = 0.049 *
Total bilirubin, µmol/L	M ± SD 189.88 ± 89.33	171.78; 207.97	M ± SD 155.13 ± 90.54	139.89; 170.37	*p* = 0.004 *
AST, U/L	36.00	31.00; 49.75	32.00	22.00; 46.00	*p* = 0.008 *

* Statistically significant differences at *p* < 0.05. RDS—respiratory distress syndrome; CP—congenital pneumonia; RBC—red blood cell count; SpO_2_—saturation peripheral oxygen; pCO_2_—partial pressure of carbon dioxide; AST—aspartate aminotransferase; Me—median; Q1; Q3—lower and upper quartiles; M—mean; SD—standard deviation.

**Table 2:** Predictors of statistically significant associations in congenital pneumonia (CP).

Dependent Predictors	Association Predictors *
Severe Condition	Weak cry (Cramer’s V = 0.48, OR = 0.099, 95% CI: 0.041–0.236, *p* < 0.001) Cough (Cramer’s V = 0.17, OR = 0.339, 95% CI: 0.136–0.845, *p* = 0.017) Weak breathing (Cramer’s V = 0.42, *p* = 0.008) Rales (Cramer’s V = 0.43, OR = 6.74, 95% CI: 3.101–14.6, *p* = 0.001) Concurrent cerebral depression (Cramer’s V = 0.36, OR = 6.70, 95% CI: 2.604–17.280, *p* = 0.001) Complications (Cramer’s V = 0.56, OR = 19.77, 95% CI: 7.112–54.97, *p* < 0.001) Length of hospital stay (Me = 17.00, Q1; Q3 [13.00; 28.50], *p* = 0.001) Heart rate (Me = 136.00, Q1; Q3 [122.00; 148.00], *p* = 0.001) Pathological changes on neurosonography (Cramer’s V = 0.28, OR = 4.03, 95% CI: 1.592–10.235, *p* = 0.002) Bacteremia (Cramer’s V = 0.22, *p* = 0.004) Mechanical ventilation (MV) (Cramer’s V = 0.67, OR = 131.38, 95% CI: 16.96–1017.81, *p* < 0.001)
Mechanical Ventilation (MV)	Hemoglobin level (OR = 29.92, 95% CI: 20.27–39.56, *p* < 0.001) Lymphocytes (*p* < 0.001) Rales (Cramer’s V = 0.51, OR = 13.509, 95% CI: 5.164–35.135, *p* = 0.001) Dyspnea, cough (Cramer’s V = 0.15, OR = 0.275, 95% CI: 0.103–0.735, *p* = 0.019) Severe condition on admission (Cramer’s V = 0.67, OR = 131.385, 95% CI: 16.959–1017.810, *p* < 0.001) Complications affecting various organs/systems (Cramer’s V = 0.40, OR = 13.163, 95% CI: 3.783–45.802, *p* < 0.001) Cerebral depression (Cramer’s V = 0.28, OR = 6.59, 95% CI: 1.893–22.950, *p* = 0.001)
Weak Cry (Stuporous State)	Total bilirubin (*p* < 0.001) Monocyte changes (*p* = 0.021) Lymphocytes (*p* < 0.001) Eosinophils (*p* = 0.049) Platelets (*p* < 0.001) Erythrocytes (*p* < 0.001) Decreased hemoglobin (MD = 25.27, 95% CI: 32.71–17.83, *p* < 0.001)
Neonatal Reflex Depression	Complications (Cramer’s V = 0.30, OR = 15.830, 95% CI: 1.988–126.066, *p* = 0.01) CBC (decreased erythrocytes, hemoglobin, thrombocytopenia, lymphopenia) (*p* = 0.002) sO_2_ (%) (Me = 85.6, Q1; Q3 [81.35; 88.70], *p* = 0.005) Lactate (Me = 1.7, Q1; Q3 [1.40; 2.80], *p* = 0.048) Glucose, mmol/L (Me = 5.60, Q1; Q3 [4.85; 6.95], *p* = 0.003)
Muscle Hypotonia	Birth weight (M ± SD = 2517.54 ± 849.08, MD = 402.91, 95% CI: 119.26–686.56, *p* = 0.006) Monocytes (Me = 0.91, Q1; Q3 [0.64; 1.62], *p* = 0.002) Lymphocytes (M ± SD = 4.61 ± 22.42, MD = 1.10, 95% CI: 0.20–2.10, *p* = 0.031) Decreased hemoglobin (M ± SD = 143.44 ± 32.93, MD = 12.29, 95% CI: 1.24–23.34, *p* = 0.030)
Retinopathy	Neonatal jaundice (*p* = 0.02) Birth weight (M ± SD = 2330.78 ± 733.50, MD = 918.31, 95% CI: 695.20–1141.43, *p* = 0.048) Transfer to NICU (Cramer’s V = 0.46, OR = 23.929, 95% CI: 5.434–105.372, *p* = 0.01) Cerebral ischemia (Cramer’s V = 0.16, OR = 2.929, 95% CI: 0.991–4.101, *p* = 0.052)
Surfactant Therapy	Cerebral ischemia (Cramer’s V = 0.25, OR = 12.533, 95% CI: 1.628–96.504, *p* = 0.001) Retinal changes (Cramer’s V = 0.31, OR = 8.077, 95% CI: 2.260–28.871, *p* = 0.001) Gestational age (Me = 34.00, Q1; Q3 [33.00; 35.00], *p* = 0.002) Organic brain pathology (e.g., hemorrhages and ventricular dilation) (Cramer’s V = 0.37, *p* = 0.001) Decreased hemoglobin (M ± SD = 132.90 ± 26.65, MD = 21.26, 95% CI: 8.38–34.15, *p* = 0.001)
Dyspnea and Cough	Severe condition on admission (Cramer’s V = 0.20, OR = 0.339, 95% CI: 0.136–0.845, *p* = 0.052) Weak cry (Cramer’s V = 0.17, OR = 2.190, 95% CI: 0.992–4.833, *p* = 0.050) Accessory muscle use (Cramer’s V = 0.39, OR = 8.250, 95% CI: 3.071–22.160, *p* = 0.001)
Pathological Heart Murmurs	CBC (erythrocytes, M ± SD = 3.93 ± 1.01, MD = 0.51, 95% CI: 0.09–0.94, *p* = 0.019) Hemoglobin (M ± SD = 134.34 ± 30.84, MD = 19.10, 95% CI: 4.84–33.36, *p* = 0.009)
Changes in Heart Sounds	Duration of hospital stay (*p* = 0.003) Mechanical ventilation (Cramer’s V = 0.46, OR = 0.097, 95% CI: 0.039–0.243, *p* = 0.001)

* Cramer’s V—a measure of the strength of association between two categorical variables. MV—mechanical ventilation; CBC—complete blood count; NICU—neonatal intensive care unit; Me—median; Q1; Q3—lower and upper quartiles; M—mean; MD—mean difference; OR—odds ratio; CI—confidence interval.

**Table 3:** Predictors of statistically significant associations in respiratory distress syndrome (RDS).

Dependent Predictors	Association Predictors *
Severe Condition	Length of hospital stay (Me = 19.50, Q1; Q3 [13.25; 27.75], *p* = 0.001) Heart rate (Me = 138.00, Q1; Q3 [128.75; 144.50], *p* = 0.001)
Mechanical Ventilation (MV)	pCO_2_ (M ± SD = 43.42 ± 6.23, MD = −8.27, 95% CI: −15.09 to −0.58, *p* = 0.036) Concurrent cardiovascular diseases (Cramer’s V = 0.26, OR = 7.111, 95% CI: 1.349–37.486, *p* = 0.016)
Retinal Changes	Cerebral ischemia (Cramer’s V = 0.20, OR = 2.302, 95% CI: 1.021–5.190, *p* = 0.043) Gestational age (Me = 35.00, Q1; Q3 [34.00; 36.00], *p* = 0.001)
Surfactant Therapy	Low gestational age (Me = 33.50, Q1; Q3 [32.00; 35.70], *p* = 0.001)

* Cramer’s V—a measure of the strength of association between two categorical variables. MV—mechanical ventilation; pCO_2_—partial pressure of carbon dioxide; Me—median; Q1, Q3—lower and upper quartiles; M—mean; MD—mean difference; OR—odds ratio; CI—confidence interval.

**Table 4:** Multivariable Firth’s penalized logistic regression model for differential diagnosis (CP vs. RDS).

Predictor	β	SE	OR	95% CI	*p*-Value
Gestational age (weeks)	−0.35	0.08	0.70	0.60–0.82	<0.001
Birth weight (g)	0.001	0.0003	1.001	1.000–1.002	0.042
Hemoglobin (g/L)	−0.02	0.007	0.98	0.96–0.99	0.015
Cerebral ischemia	1.15	0.38	3.16	1.50–6.67	0.002

Dependent variable: diagnosis (CP = 1, RDS = 0). Firth’s penalized logistic regression was used to reduce small-sample bias and to obtain stable odds ratios. CI—confidence interval; SE—standard error; OR—odds ratio. Only predictors with stable estimates are shown.

**Table 5:** Performance metrics of the logistic regression and Random Forest models on the independent test set (20% holdout).

Metric	Firth’s Logistic Regression	Random Forest
Accuracy	0.66	0.69
Sensitivity	0.60	0.62
Specificity	0.68	0.74
PPV	0.55	0.60
NPV	0.72	0.75
F1-score	0.57	0.61
ROC-AUC	0.76	0.82
Brier score	0.20	0.18

Abbreviations: PPV—positive predictive value; NPV—negative predictive value; ROC-AUC—area under the receiver operating characteristic curve. Metrics were calculated on the independent test set (20% of the data, *n* = 49), which was not used during model training or hyperparameter tuning.

## Data Availability

The data presented in this study are available upon request from the corresponding author due to privacy and ethical restrictions.

## Abbreviations

The following abbreviations are used in this manuscript:

RDS	Respiratory distress syndrome
CP	Congenital pneumonia
MV	Mechanical ventilation
ICU	Intensive care unit
AST	Aspartate aminotransferase
AUC	Area under the curve
BW	Birth weight
ELBW	Extremely low birth weight
M	Mean
SD	Standard deviation
CI	Confidence interval
Me	Median
MD	Mean difference
Q1; Q3	Lower and upper quartiles
OR	Odds ratio
RBC	Red blood cells
SpO_2_	Saturation peripheral oxygen
pCO_2_	Partial pressure of carbon dioxide
CNS	Central nervous system
CBC	Complete blood count
VSD	Ventricular septal defect
ASD	Atrial septal defect
PDA	Patent ductus arteriosus
NICU	Neonatal intensive care unit
TSH	Thyroid-stimulating hormone
